# Amylic and Ethylic Alcohol Differentiated; Physiological Action and Therapeutical Uses and Abuses of the Alcohol Group; Education as Essential as Legislation in Solving the Alcohol Problem

**Published:** 1910-08

**Authors:** B. W. Hall

**Affiliations:** Bowman, Ga.


### AMYLIC AND ETHYLIC ALCOHOL DIFFERENTIATED; PHYSIOLOGICAL ACTION AND THERAPEUTICAL USES AND ABUSES OF THE ALCOHOL GROUP; EDUCATION AS ESSENTIAL AS LEGISLATION IN SOLVING THE ALCOHOL PROBLEM.

BY B. W. HALL, A. B., M. D., BOWMAN, GA.

Medical names: Ethyl, Ethylic, Absolute Alcohol, Ethyl Hydrate, Alcohol Ethylicum.

Chemical Formula: C2, H5, OH.

Alcohol is a colorless volatile liquid of aromatic odor, being distilled from vinous fermentation. It is composed of 91% by weight (94% by volume) of water. Sp. Gr. 0.82 at 15.6 % C. (60 F.). Dilute alcohol is composed of 45.5% by weight (53% by volume) of ethyl alcohol and 54.5% by weight (47% by volume) water. When grain or potato whisky is distilled for the purpose of obtaining alcohol the pure spirit will continue to come over for a certain time. Now, place oyster shells, charcoal, sand bed, or a few drops of olive oil in the proper place in the condensation and continue the distillation a little further, a milky liquid will appear, which upon standing will be covered with a stratum of this peculiar oil. Now, subject the milky liquid to distillation. It will at first boil at a comparatively low temperature and yield water and a little of the oil, but after a time the boiling point will rise to 132 C. (269 F.) When the *oil will come over pure.* By changing the receiver when the oil begins to distill free from water, the oil is collected separately.

Amylic alcohol C5, H11, HO is an oily colorless liquid of strong offensive odor and acrid, burning taste. It boils at 132 (269 F.) Sp. Gr. 0.81. It is slightly soluble in water and unites in all proportions with alcohol, ether and essential oils. It dissolves iodine, sulphur and phosphorus and is a good solvent for fats, resins and camphor. Amylic alcohol C5, H11, HO, when subjected to oxidizing agents, it loses two aloms of hydrogen and gains one of oxygen, and becomes C5, H10, O2, or amylic acid, the acid found in valerian.

The free amyl (C5, H11) 2 has been isolated. Its hydride

$C_5, H_{11}, H$ has active anasthetic properties discovered by Dr. Simpson, of Edenburg, Scotland. Crude fusil oil is an active irritant poison and is composed of prophylic butylic=$C_7, H_{10}, O$, and amylic alcohol. It is largely used in the manufacture of cinchona alkaloids and in the arts. Methyl, methylic alcohol= $C, H_4, O$, is wood alcohol, which is also used in the fine arts.

Ethyl alcohol, in small doses, is used internally as a cardiac stimulant in debilitating diseases: diphtheria, typhoid fever, pneumonia, nervous collapse and surgical shock. It is also used locally as an antiseptic, etc., and astringent, and for the preservation of anatomic and biologic specimens, for the preserving and making of medical tinctures and fluid extracts. In large doses alcohol is a narcotic poison, producing intoxication with muscular inco-ordination, delirium and coma.

### ALCOHOLIC ABUSE.

Recent competent observers show that the tissue, brain, nervous system, heart, blood vessels, the stomach, intestinal tract, the lymphatic system, the kidneys, may all become diseased by the habitual drinking of even small quantities of alcohol. The circulation of alcohol into the blood tends to attack the weakest organ or tissue. It is generally conceded that alcohol is a poison and its increase of muscular power is not supported by facts. Its use even in small quantities causes a decrease in at least 10% of muscular power and energy. The observations made by Schumburg, Scheffer, Abel, Smith and others do not prove that alcohol is a food in the sense in which fats and carbohydrates are food. It should be defined as an easily oxidizing agent with numerous untoward effects which inevitably appear when certain minimum doses are exceeded. It should be classed with the more or less dangerous stimulants and narcotics, such as hashish, tobacco, etc., rather than with truly sustaining food stuffs. The experiments of German investigators prove beyond question that alcohol is a disturber rather than promoter of brain activity. The effects of alcohol is cumulative, so that the drinker of a single bottle of wine daily is in reality never actually sober, even though wine in itself is not a poison. Wine causes a loss of at least 10% in working efficiency and aids the action of other poisons, such as those of contagion. The dissecting knife frequently reveals post-mortum, a "hobnail liver" or an alcoholic kidney, stomach, heart, or watery alcohol brain. At least one-third of pauperism, 50% of the orphanage inmates, one-fourth of the unfortunates in insane asylums, half of the convicts in our prison wards, four-fifths of the inmates of our jails and work-houses are thought to be due to alcohol, as is a large percentage of suicides, sudden deaths and venereal diseases and a large proportion of marital infelicity.

The insane, the criminals of various types, and the recipients of charity make up the great mass of abnormal members of the body politic whose unfitness receives official recognition. For every individual that dies prematurely of a disease directly due to alcohol, there are scores of individuals that suffer to a lesser degree from maladies which are wholly or in part of the same origin, but which are not directly fatal. For every patient that suffers complete mental collapse as the result of alcoholism there are scores of patients that are victims of epilepsies, neurasthenias, neuralgias, choreas and palsies of alcoholic origin. For every criminal that alcohol sends to prison there are scores of persons whose moral delinquencies induced or emphasized by alcohol are not of the indictable order, yet are a source of suffering to their friends and a detriment to humanity. For every incapable who, weakened by alcohol, acknowledges defeat in life's battle and openly seeks alms, there are scores of individuals that feel the pressure of want in greater or less degree because the money that might have supplied the necessities and luxuries has gone to drink, yet they strive to hide their indigence. But all these are without the field of statistics. They have no share in the estimates presented. As we view this joyless pageant, the vast majority of its members impelled by a power they loathe yet must obey, a realizing sense comes to us of the tyranny exercised over humanity, generation after generation, by this arch enemy of progress.

### ALCOHOL AS AN ETIOLOGICAL FACTOR IN CANCER.

Much speculation as to the cause of cancer fill our medical journals. The germ theory seems to be falling into insignificance giving place to the more rational cause of high living, premature decay and alcoholic excess. The amount of damage done to individual takers of alcohol and to communities as a whole cannot be fully estimated. The amount of evil done by taking regularly small quantities into the tissues of the body is not generally recognized. Alcohol by a powerful affinity for water of the tissue of the body dehydrates and prematurely hardens them.

Alcohol is a retarder of waste, diminishing the metamorphosis of tissue, hindering the separation from the tissues of the body of those effete and waste products which should be eliminated. The waste matters are retained in the body and tissue-hardening and degeneration of organs are the result. This does not apply to the drunkard, as we all know what his fate will be. Many persons above suspicion live daily under the influence, and die from the effects of alcoholic drinks, who are never suspected of using them. The daily use at meals of various bitters, etc., is essentially nothing more than a thinly disguised tippling under the form of medication, and produces dire effects in the course of time, especially when at the same time little or no bodily exercise is taken. Hardening and degenerative changes caused by alcohol upon the tissue of the body sometimes result in local irritation, an injury, neucrosis of the living tissue, a malignant or other neoplasm may result

ALCOHOL AS AN ETIOLOGICAL FACTOR IN TUBERCULOSIS.

Mr. Jacques Bertillon has produced a set of maps, French statistics: "The first may shows where the culture of the vine is greatest and where most alcohol is consumed tuberculosis is greatest, with some few exceptions. The phthisis map be superimposed on the alcohol map. On the other hand phthisis is more frequent among saloon-keepers than with other merchants (579 deaths annually in 100,000 persons as compared with 245). It is probable alcohol also that makes phthisis twice as frequent in Paris among men than among women."

Polar expeditions have proven beyond contradiction that alcohol lowers the resisting power of the human body. That alcohol decreases the resisting power of the human body is further evidenced by a larger death rate of alcoholics in pneumonia, typhoid fever, and other infectious diseases.

### NATIONAL INEBRIETY.

The Country Life Commission appointed by president Roosevelt made some startling discoveries regarding the alcohol problem the publication of which was supported by the government. Alcohol addicts develop a nation of runts and inebriates. In the waning nations of the world it is estimated that there are 2,000,000 child drinkers or child alcoholist. Norway and Sweden being the only nations in Europe exempt from child alcoholism. In London alone there are 300,000 child drinkers or about 40% of all children. In New York the per centage of child drinkers is 58%.

### DECREASED CONSUMPTION OF ALCOHOL.

The per capita consumption of liquor in 1850 was about three gallons. In 1880 it was fifteen gallons, including spirits, beer and wine. The consumption scarcely equalling the increase in population. The increase per capita in 1880 was due to a considerable degree to increased immigration. It is gratifying to know that fully 40,000,000 of the population of the United States are now in dry territory. Nine States: Maine, Kansas, North Dakota, North Carolina, Georgia, Alabama, Mississippi, Tennessee and Oklahoma are dry. For the first time in the history of prohibition reform there is real and marked decrease in the consumption of alcoholic liquors.

For the year ending June 30, 1908, according to figures of the United States internal revenue office, the production of distilled liquors was 126,989,974 gallons. The year previous ending June 30, 1907, the production was 168,573,915 gallons, which indicates a decrease in 1908 of over forty-one million gallons.

President Liebman, at a meeting of the National Brewers' Association, held at Atlanta City recently, declared that there had been a falling off in the manufacture of beer in the previous 18 months amounting to 5,500,000 barrels. There is a heavy decrease in the consumption of liquor brought about by the prohibition movement. As evidence of this fact the United States revenue, through its tax on distilled and fermented liquors, decreased last year more than five million dollars. Before the new dispensation the physician was a part of the priesthood officiating in the holy of holies, in which wine was strictly forbidden to enter. The modern physician may have been shorn of his priestly prominence but he is still a teacher in the art and science of medicine and all true healing is from God.

The solution of the alcohol problem hinges as much upon education as upon legislation. The modern physician should raise the moral and religious standards of the medical profession by teaching and disseminating the facts about the alcohol problem.

The Federation Bureau of the United States was organized in 1906. It co-operates with the International Bureau of Europe in the collection and dissemination of facts upon the alcohol problem. Physicians interested in the scientific facts upon the alcoholic problem may have their names registered and receive literature and research studies of medical men by addressing the chairman of the Federation Bureau, T. D. Crothers, M. D., Hartford, Conn.

---

### SURGICAL SUGGESTIONS.

The presence of papulo-squamous tuberculides may be the only means of recognition of tuberculosis in infancy.

---

A foreign body lodged in a bronchus may present a symptom-complex identical with pulmonary tuberculosis in a child—the history of aspiration of the foreign body may be wanting.

*American Journal of Surgery.*

---

### THE CHARLOTTE SANATORIUM.

The first annual report of the Charlotte Sanatorium for the year 1909 makes a very creditable showing for this up-to-date and progressive institute. The total number of patients admitted was 839 with a death rate of 2½ per cent. The high standing of the men in charge of the hospital and the excellence of the work is assurance of continued success.

